# Which Approach Is More Effective in the Selection of Plants with Antimicrobial Activity?

**DOI:** 10.1155/2013/308980

**Published:** 2013-06-26

**Authors:** Ana Carolina Oliveira Silva, Elidiane Fonseca Santana, Antonio Marcos Saraiva, Felipe Neves Coutinho, Ricardo Henrique Acre Castro, Maria Nelly Caetano Pisciottano, Elba Lúcia Cavalcanti Amorim, Ulysses Paulino Albuquerque

**Affiliations:** ^1^Rede Nordeste de Biotecnologia (RENORBIO), Programa de Pós-Graduação, Universidade Federal Rural de Pernambuco, Rua Dom Manoel de Medeiros, s/n, Dois Irmãos, 52171-900 Recife, PE, Brazil; ^2^Departamento de Biologia, Laboratório de Etnobotânica Aplicada, Universidade Federal Rural de Pernambuco, Rua Dom Manoel de Medeiros, s/n, Dois Irmãos, 52171-900 Recife, PE, Brazil; ^3^Departamento de Biologia, Universidade de Pernambuco, Avenida Agamenon Magalhães, s/n, Santo Amaro, 50100-010 Recife, PE, Brazil; ^4^Departamento de Farmácia, Laboratório de Análises Microbiológicas, Universidade Federal de Pernambuco, Avenida Professor Moraes Rego, 1235 Cidade Universitária, 50670-901 Recife, PE, Brazil; ^5^Departamento de Farmácia, Laboratório de Produtos Naturais, Universidade Federal de Pernambuco, Avenida Professor Moraes Rego, 1235 Cidade Universitária, 50670-901 Recife, PE, Brazil

## Abstract

The development of the present study was based on selections using random, direct ethnopharmacological, and indirect ethnopharmacological approaches, aiming to evaluate which method is the best for bioprospecting new antimicrobial plant drugs. A crude extract of 53 species of herbaceous plants collected in the semiarid region of Northeast Brazil was tested against 11 microorganisms. Well-agar diffusion and minimum inhibitory concentration (MIC) techniques were used. Ten extracts from direct, six from random, and three from indirect ethnopharmacological selections exhibited activities that ranged from weak to very active against the organisms tested. The strain most susceptible to the evaluated extracts was *Staphylococcus aureus*. The MIC analysis revealed the best result for the direct ethnopharmacological approach, considering that some species yielded extracts classified as active or moderately active (MICs between 250 and 1000 *µ*g/mL). Furthermore, one species from this approach inhibited the growth of the three *Candida* strains. Thus, it was concluded that the direct ethnopharmacological approach is the most effective when selecting species for bioprospecting new plant drugs with antimicrobial activities.

## 1. Introduction

The search for new, natural compounds is growing, mainly due to the acquired resistance of microorganisms to commonly used drugs [1–3] and because nosocomial infections caused by these microorganisms have increasingly resulted in public health problems. Although several antibiotics are available on the market, microbial resistance to them has increased significantly, mainly due to the extensive use of drugs and the rapid genetic transfer of resistance. It is estimated that microbial resistance develops within seven to eight years of regular antibiotic use [2, 4]. Therefore, the development of new drugs that are capable of overcoming microbial resistance is critical.

Given these circumstances, bioprospection studies have been developed, aiming to identify plants from which new drugs may be produced, either using crude plant extracts or by isolating and characterizing the isolated active components; moreover, the goal of these investigations is to understand the role of these components as the basis for the development of new drugs (both natural and synthetic) from plants [5]. It is estimated that 30 to 40% of the most recent antimicrobial drugs available on the market are derived from natural products. However, these resources have been poorly explored to produce additional antimicrobial drugs from plants [6].

It is known that plants provide an unlimited range of compounds because of their high chemical diversity and because they have been used for centuries by several different peoples. For example, *Arctostaphylos uva-ursi* (L.) Spreng and *Vaccinium macrocarpon* Aiton are used to treat urinary tract infections, and *Melissa officinalis* L., *Allium sativum* L., and *Melaleuca alternifolia* Cheel are well-known broad-spectrum antimicrobial agents [7].

Currently, one of the major challenges is the selection of plants for a bioprospecting study because this process is the first research step. The various methods include the selection approach, which is used to choose plants based on certain criteria: random selection, which involves the arbitrary collection of the species without consideration of, for instance, taxonomic affinities and ethnobotanical information; ethnodirected selection, which includes ethnobotanical and ethnopharmacological approaches and applies information about the traditional use of plants to treat specific diseases; and the chemotaxonomic approach, which is based on the study of plants from the same family or genus of a species from which active compounds have been already isolated [8].

Several studies have been focused on selecting plants through the ethnodirected approach [1, 9, 10], corroborating the folk use of many species that are traditionally used. Several authors also suggest that random selection should be avoided in favor of ethnodirected selection because the latter appears to be a more efficient method of selecting species for bioprospecting [7].

Despite the large body of published data (namely, the studies cited previously) suggesting that ethnodirected selection is more efficient, few studies have compared the different selection approaches (e.g., see Melo [11] and Svetaz et al. [12]) to provide a more realistic scenario for bioprospecting studies.

The main aim of the present study was to assess whether the probability of discovering plants with antimicrobial potentials is greater when they already have indications of ethnopharmacological use for infectious and parasitic diseases (direct ethnopharmacological selection) than when they are randomly selected (random selection) or do not have direct indications of use for infectious and parasitic diseases (indirect ethnopharmacological selection). Furthermore, we aimed to determine the selection group to which the plants belonged with the lowest minimum inhibitory concentration (MIC), that is, to assess whether plants with higher antimicrobial activities are among those with a history of folk use in the treatment of infectious diseases. Finally, we aimed to define which species should be selected for future investigations involving compound isolation and identification for the production of antimicrobial phytopharmaceuticals.

### 1.1. Selection and Collection of Species

For species selection, a database was created from 10 floristic and/or phytosociological studies conducted in the “Caatinga” (savanna-like vegetation) in the semiarid region of Northeast Brazil. Six hundred forty-five species belonging to 81 families and 319 genera were included. Subsequently, the folk use for each species was assessed in the literature, totaling 147 species with indications for use. For the random selection, all species without medicinal uses were considered. For the direct ethnopharmacological selection, plants with indications of use for infectious and parasitic diseases, such as pleuritis, helminthiasis, general inflammation, urinary disorders, prostate infection, and uterine inflammation, were considered. For the indirect ethnopharmacological selection, all of the species with any indications of medicinal use were selected, excluding those species that were related to the direct ethnopharmacological approach. Thereafter, the Biostat 5.0 software was used for species selection. Initially, 20 species were randomly selected for each type of approach. However, due to the unavailability of certain species during the collection period, 19 species of herbs were selected for the random approach, 20 for the direct ethnopharmacological selection, and 14 for the indirect ethnopharmacological selection.

In total, 53 species of plants were collected from April to July 2011 at the Experimental Station of the Agricultural Research Company of Pernambuco, Agronomic Institute of Pernambuco (Instituto Agronômico de Pernambuco (IPA)) (8°14′S and 35°55′W, 537 m altitude) in Caruaru, Agreste region of Pernambuco, Northeast Brazil. Voucher specimens are deposited in the Herbarium of Professor Vasconcelos Sobrinho of the Federal Rural University of Pernambuco (PEUFR). The area was selected because it is well studied in terms of floristics and provides easy access for obtaining the various species.

### 1.2. Extract Preparation

Because the species were herbaceous, all of the extracts were prepared from the leaves. Twenty grams of dried and powdered leaves was macerated in absolute methanol for 24 hours. The process was extensively repeated. The extract obtained was then evaporated under reduced pressure at 45°C until dried. The samples were placed in a desiccator for a one-week period. The extracts were diluted in 20% dimethyl sulfoxide (DMSO) and used at concentrations of 100 mg/mL and 50 mg/mL. As there is no standard regarding the maximum concentration of crude extract of plants for screening, the protocol of the Laboratory of Microbiological Analyses, from Federal University of Pernambuco was adopted, where the assays were performed.

### 1.3. Antimicrobial Assay

The antimicrobial activity was evaluated in two trials. First, the antimicrobial activity was determined by the agar diffusion method [13]. Next, for the species that displayed inhibition halo greater than 13 mm, the MIC [13] was determined.

The crude extracts were tested against standard strains of *Staphylococcus aureus* (ATCC 6538), *S. epidermidis* (sperm), *S. saprophyticus* (LACEN 07), *Bacillus subtilis* (ATCC 6633), *Enterococcus faecalis* (ATCC 51299), *Klebsiella pneumoniae* (secretion), *Pseudomonas aeruginosa* (ATCC 14502), *Escherichia coli* (ATCC 35218), *Candida albicans* (urine), *C. krusei* (blood), and *C. tropicalis* (rectal swab). These strains were selected because of their clinical relevance.

Inoculums were prepared 24 hours in advance and kept in Mueller-Hinton agar (bacteria) and Sabouraud agar (yeast). The inoculums were suspended in sterile saline solution using a 0.5 McFarland standard (10^8^ UFC/mL) [14].

For the agar diffusion method, sterile swabs were used to inoculate sterile petri plates (20 × 100 mm) containing 20 mL of Mueller-Hinton agar for bacteria and 20 mL Sabouraud agar for yeast. On each plate, four wells (6 mm diameter) were created, to which 100 *μ*L of the extracts at concentrations of 100 mg/mL and 50 mg/mL was added, as well as the positive controls tetracycline (30 mg/mL) for bacteria and ketoconazole (50 mg/mL) for yeast. Twenty percent DMSO was used as the negative control. The plates were incubated aerobically at 37 ± 1°C for 24 hours. The antimicrobial activity was assessed by measuring the inhibition halo of microbial growth around the well, and the results were classified according to the following scale: inhibition zones down to 9 mm, inactive; 9–12 mm, moderately active; 13–18 mm, active; above 18 mm, very active [15]. The *G*-test was performed (*P* < 0.05) to determine significant differences between the selection approaches and the antimicrobial activity. All of the assays were performed in triplicate.

The MIC of the extracts and the reference antibiotic (tetracycline) were determined using the Mueller-Hinton broth microdilution technique following the protocol established by the Clinical and Laboratory Standards Institute [16] for bacteria. Inoculums were prepared in the same medium and were adjusted to a 0.5 McFarland standard (10^8^ UFC/mL) and diluted 1 : 10 for the broth dilution method. The microplates were incubated at 37°C, and the MIC was read after a 24-hour incubation period. The MIC was defined as the lowest compound concentration at which the microorganism tested showed no visible growth.

The MIC for yeasts was performed by the broth microdilution technique according to the CLSI [17], and ketoconazole was used as the positive control. The inoculum was used at a concentration of 1.0 × 10^6^ UFC/mL. The MIC was determined in Roswell Park Memorial Institute (RPMI) 1640 medium (Gibco, Invitrogen Co., New York, USA), with MOPS buffer, pH 7.0. The plates were incubated at 37°C, and the readings were obtained after a 24-hour incubation period. All of the assays were performed in duplicate.

Plant extracts with MICs <100 *μ*g/mL were considered highly active antimicrobial agents; MICs ranging from 100 to 500 *μ*g/mL were classified as active; MICs ranging between 500 and 1000 *μ*g/mL were considered moderately active; MICs ranging from 1000 to 2000 *μ*g/mL were considered to have low activity; and MICs >2000 *μ*g/mL were classified as inactive [14]. The *G*-test was conducted (*P* < 0.005) to evaluate significant differences between the selection approaches and the MIC.

## 2. Results and Discussion

Of the 20 plant extracts obtained by direct ethnopharmacological selection, 10 (50%) exhibited activity against the microorganisms tested. Of the 19 plant extracts obtained by random selection, seven (36.84%) showed antimicrobial activity against at least one strain. Of the 14 plant extracts obtained by indirect ethnopharmacological selection, two (14.28%) exhibited antimicrobial activity (Table 1). The active extracts that were tested exhibited antimicrobial activity only against the Gram-positive bacteria and the *Candida* strains.

Of the 10 extracts obtained by direct ethnopharmacological selection, 40% exhibited moderate antimicrobial activity, 30% were active, and 30% were highly active. Of the extracts obtained by random selection, 33.33% exhibited moderate activity, and 66.66% were highly active. Of the extracts obtained by indirect ethnopharmacological selection, 70% were moderately active, and 30% were active. Based on the *G*-test (*G* = 127.1860), significant differences (*P* < 0.005) existed between the proportions of active species among the three approaches, indicating that direct ethnopharmacological selection is the most effective for the selection of plants with greater antimicrobial activities.

The plant species obtained by direct ethnopharmacological selection were more effective in terms of the number of strains with inhibited growth. Four of the plants, *Acanthospermum hispidum*, *Euphorbia hyssopifolia*, *Hyptis suaveolens*, and *Indigofera suffruticosa*, displayed antimicrobial activity against four microorganisms. Two of the plants, *Ludwigia octovalvis* and *Momordica charantia*, were active against five microorganisms. *Centratherum punctatum*, which was obtained by random selection, inhibited the growth of five microorganisms, whereas *Blainvillea acmella* inhibited four microorganisms. Only *Tillandsia recurvata*, which was obtained by indirect ethnopharmacological selection, displayed antimicrobial activity against five microorganisms.

Although the plant species that were selected by the three approaches exhibited the same versatility in the inhibition of various strains (i.e., five microorganisms from the 11 that were tested), the selection approaches were distinguished by the antimicrobial activities presented by the species; this activity was greater in the species obtained by direct ethnopharmacological selection, resulting in the largest inhibition halo (ranging from 22 to 30 mm). Furthermore, of the 53 plant species studied, only four of the extracts could inhibit five microorganisms; two of these plant species were obtained by direct ethnopharmacological selection. The findings of the present study indicate greater success when plant species are selected based on their direct indications of use for infectious and parasitic diseases, which is similar to the findings of Phongpaichit et al. [18], who investigated the use of plants to treat fungal infections in AIDS patients. By selecting species based on folk use, the authors achieved a success rate of 40%. Cruz et al. [9], analyzing Brazilian plants traditionally used to treat mycoses, achieved a success rate of 50%, confirming the results of the present study. van Vuuren and Naidoo [10] analyzed plants (selected from the ethnobotanical literature) used in the treatment of sexually transmitted diseases and found that 90% of the extracts exhibited significant antimicrobial activity against the tested microorganisms, thereby validating the ethnomedicinal use of species. Comparing the results obtained from the different selection approaches (Table 1), direct ethnopharmacological selection yielded a greater number of species that were active against the tested microorganisms—plants such as *Acanthospermum hispidum*, *Euphorbia hyssopifolia*, and *I. suffruticosa*, which caused inhibition halo ranging from 17 to 30 mm for the *S. aureus*, *S. epidermidis*, *S. saprophyticus*, and *B. subtilis* strains. Another example of the efficiency of plant species obtained by direct ethnopharmacological selection was their action against *E. faecalis*. Whereas three of the plant species were able to inhibit the growth of *E. faecalis*, only one species obtained by random selection and one species obtained by indirect ethnopharmacological selection inhibited this microorganism.

The MIC was determined for four species obtained by random selection, nine species obtained by direct ethnopharmacological selection, and two species obtained by indirect ethnopharmacological selection: *Blainvillea acmella*, *Centratherum punctatum*, *Sida urens*, and species 1 that is in process of patent registration, and so its name cannot be disclosed, (random selection); *Acanthospermum hispidum*, *Argemone mexicana*, *E. hyssopifolia*,* Hyptis suaveolens*, *I. suffruticosa*, *Leonotis nepetifolia*, *Ludwigia octovalvis*, *Melochia tomentosa*, and *Momordica charantia* (direct ethnopharmacological selection); and *Tillandsia recurvata *and *Hypenia salzmannii* (indirect ethnopharmacological selection). The MICs for all of the analyzed species and strains are presented in Table 2. Based on the *G*-test (*G* = 76.5443), a significant difference (*P* < 0.005) existed among the MICs of the three selection approaches, with direct ethnopharmacological selection again being distinct because extracts of plant species belonging to this group exhibited superior MICs. The extracts were tested only against the strain to which they showed activity up to 13 mm.

All of the species obtained by indirect ethnopharmacological selection were considered to have low activities because they displayed MICs above 1000 *μ*g/mL. In the random selection, only *Sida urens* was considered moderately active, presenting an MIC of 500 *μ*g/mL against *S. aureus*. For species obtained by direct ethnopharmacological selection, four extracts were classified as moderately active: *A. hispidum*, *E. hyssopifolia, I. suffruticosa*, and *Momordica charantia*. *Ludwigia octovalvis* from the direct ethnopharmacological selection and species 1 from the random selection were the only species with MICs that were classified as active and highly active for *C. albicans* (125 *μ*g/mL and 31.25 *μ*g/mL, resp.). *L. octovalvis* also exhibited a moderately active extract against *C. krusei* and *C. tropicalis* (1000 *μ*g/mL), while the extract from species 1 was highly active against *C. krusei* (62.5 *μ*g/mL) and *C. tropicalis* (62.5 *μ*g/mL). According to Fabry et al. [19], plants with MICs below 8 mg/mL are considered to display some antimicrobial activity. Moreover, our findings indicate that these species are potential candidates for further investigations (of the isolation and identification of compounds with antimicrobial activities) because the MICs were below 1 mg/mL, confirming other studies indicating that plant extracts and natural products with MICs below 1 mg/mL deserve special attention and must therefore be carefully analyzed [7, 20].

The results obtained in the present study indicate that direct ethnopharmacological selection is an effective bioprospecting tool for antimicrobial activity. Svetaz et al. [12] evaluated ethnomedical information on the discovery of plants with antifungal activity and determined that the probability of finding plants with this activity is significantly higher when reports exist of their use as antifungal agents compared with the absence of such reports. The authors categorically affirmed that the ethnopharmacological approach is useful in detecting plants with antifungal activity. Furthermore, in studies on the potential antimalarial effect of Nigerian plants, Adebayo and Krettli [21] discussed the difficulty, high cost, and low efficacy of the random selection approach, which was the selection method that had been used in that country decades prior. The current method is ethnobotanical selection, based on information regarding indigenous uses of the species, which has reduced costs and time compared with random selection.

The low probability of finding promising plants for bioprospection through random selection suggests that this approach is not recommended for the discovery of new antimicrobial agents. Although this approach was responsible for the discovery of taxol [22, 23], it is currently known that only one in 10,000 plants will be a promising source of new drugs, while the ethnodirected selection is responsible for 74% of all the drugs of plant origin [24]. Nevertheless, controversy exists regarding the efficacy of this approach because the selected plants appear not to be effective in the treatment of cancer [11] and mycoses caused by yeasts and *Aspergillus* spp.; indeed, for these types of fungi, Svetaz et al. [12] found no significant differences between the antimicrobial activities of plants obtained by random selection versus ethnopharmacological selection. Moreover, the fact that in the present study, only one species obtained by random selection was highly active against the three *Candida* strains indicates that the random approach should not be completely abandoned but should instead be adapted to other selection approaches, such as chemosystematic or ecological methods.

The species that were obtained by direct ethnopharmacological, random, and indirect ethnopharmacological selections were active only against Gram-positive bacteria. The inactivity against Gram-negative bacteria might be observed because of the lipophilic outer layer, among other reasons, as this layer most likely prevents the access of the extract to the interior of the bacteria, as observed by Nantitanon et al. [25] in their assessment of the antimicrobial activity of *Hyptis suaveolens*. Regarding antifungal activity, it is noteworthy that most of the studies were conducted using *C. albicans* [26]. The exceptions were the studies by Cruz et al. [9], who tested the use of plants from the Caatinga against *C*. *guilliermondii, *and De Toledo et al. [27], who tested the use of plants against *C. parapsilosis* strains. The present study also included *C. krusei* and *C. tropicalis*, which are species that are more resistant to commonly used drugs. Among the species that we investigated, *L. octovalvis* and species 1 inhibited the growth of the three *Candida* species, with the most active MICs ranging from 62.5 *μ*g/mL to 31.25 *μ*g/mL. This finding of the present study is of great importance, considering the high incidence of *C. albicans* (70%) and *C. tropicalis* (20%) in Latin America [28], the emergence of other *Candida* species resistant to antifungal agents, and, mainly, the limited number of drugs available to treat fungal infections [29].

Due to the cosmopolitan nature of the species analyzed herein, studies on the antimicrobial activity involving some of these species have been conducted in various regions worldwide, such as *Ageratum conyzoides* in Malaysia [30], which is used against cough and has displayed an MIC of 1600 *μ*g/mL for the *Mycobacterium* strains. However, in the present study, all of the tested strains were resistant to the extract from *A. conyzoides*. 

Gachet et al. [31], who tested plants traditionally used against leishmaniasis in Ecuador, found that the extract of *S. dulcis* is effective against axenic amastigotes of *L. donovani*. In the present study, this species displayed moderate activity against *S. aureus* and *B. subtilis*. Wiart et al. [26] tested the methanol extract in an antimicrobial screening of plants from Malaysia including, among others, *A. conyzoides*, *C. hirtus*, *E. prostrate,* and *H. suaveolens*; these species are effective against various microorganisms such as *B. cereus*, *P. aeruginosa*, *B. subtilis*, *S. aureus*, and *C. albicans*. In the present study, however, except for *H. suaveolens,* which inhibited the growth of *S. aureus*, *S. saprophyticus*, *S. epidermidis,* and *B. subtilis* (resulting in inhibition halo between 15 and 18 mm), the other species displayed no activity against any of the analyzed microorganisms.

Matsuse et al. [32] studied plants from Panama with potential antiviral activity and found that both the crude extract and isolated compounds from *E. hyssopifolia* were effective against HIV. In the present study, this species is one of the most effective with an MIC of 1000 *μ*g/mL for *S. aureus*, which qualifies it as a potential candidate for studies aiming to develop drugs obtained from plants of the Caatinga that are more effective against resistant strains. In addition, this finding supports the folk use of this species against microbial infections in the semiarid region of Northeast Brazil [33].

In Brazil, especially the Caatinga, few studies exist on antimicrobial activity based on ethnobotanical data, and investigations are almost nonexistent regarding species from the Caatinga, whether native or spontaneous. Cruz et al. [9] studied the extract of *Ziziphus joazeiro *Mart., *Caesalpinia pyramidalis *Tul., *Bumelia sartorum* Mart., and *Hymenaea courbaril* L., which are traditionally used to treat mycoses and found that *Z. joazeiro *and *C. pyramidalis* display significant antifungal activities, making them potential candidates for the development of new strategies to treat fungal infections.

Almeida et al. [1], in a study comparing the antimicrobial efficiency of species selected in the Caatinga and Atlantic Forest, tested the crude extract of *B. diffusa*, which displayed moderate activity against *S. aureus*, *Streptococcus faecalis*, and *Mycobacterium smegmatis* when collected in the Caatinga. However, the extract of the species from the Atlantic Forest exhibited no activity against any microorganism. In the present study, all of the tested strains were resistant to the extract of this plant. The authors concluded that the Caatinga region appears to be a promising source in the search for new compounds of plant origin, due to the larger size of the inhibition halo generated when using extracts from these species and due to their ability to inhibit a greater number of microorganisms.

Despite the fact that species 1 from the random selection displays an MIC that classifies it as highly active (≤62.5 *μ*g/mL) for the three *Candida* species tested, the findings in the present studies regarding species obtained by direct ethnopharmacological selection (i.e., *Acanthospermum hispidum*, *Euphorbia hyssopifolia*, *I. suffruticosa*, *Ludwigia octovalvis*, and *Momordica charantia, *which exhibited MICs ranging from 250 to 1000 *μ*g/mL) indicate that this selection approach is an effective strategy for bioprospecting new drugs with antimicrobial activity. Furthermore, additional in-depth studies should be conducted using compounds isolated from the cited species. The present study included only spontaneous herbaceous species of the Caatinga, which is a practice that is still undervalued in bioprospecting but that, based on our findings, appears to be potentially useful in the search for compounds with antimicrobial activity; indeed, the plant extracts used were able to inhibit yeasts that occur with high incidence in Latin America and that exhibit high resistance to regular antibiotics. Additionally, these species are characterized by wide distribution, high population numbers, and rapid growth, which would facilitate their study. Our findings are supported by the investigations of Cruz et al. [9] and Almeida et al. [1] in considering the Caatinga and ethnopharmacological selection in the search for new pharmaceutical products.

## 3. Conclusions

It may be concluded that direct ethnopharmacological selection is an important bioprospecting tool and that the Caatinga is a type of vegetation that should be included in future studies on the bioprospection of new antimicrobial plant drugs. Additionally, the above-mentioned species should be included in the studies investigating the production of new phytomedicines.

## Figures and Tables

**Table 1:** Antimicrobial activity of herbaceous plants from the semiarid region, Northeast of Brazil, based on random, direct, and indirect ethnopharmacological approaches. (Inhibition halum in mm).

Species	*C*. mg/mL	Sa	Se	Ss	Bs	Ef	Ec	Kp	Pa	Ca	Ck	Ct
Random approach												
*Astraea lobata *(L.) Klotzsch	100 50	—	—	—	—	—	—	—	—	—	—	—
*Blainvillea acmella* (L.) Philipson	100 50	23 18	20 13	20 16	22 20	—	—	—	—	—	—	—
*Centratherum punctatum* Cass.	100 50	30 25	20 17	22 14	21 16	22 14	—	—	—	—	—	—
*Croton hirtus *L'Hér.	100 50	—	—	—	—	—	—	—	—	—	—	—
*Cyperus uncinulatus* Schrad. ex. Nees	100 50	—	—	—	—	—	—	—	—	—	—	—
*Delilia biflora* (L.) Kuntze	100 50	—	—	—	—	—	—	—	—	—	—	—
*Drymaria cordata *(L.) Willd. ex Roem. & Schult.	100 50	7	—	—	—	—	—	—	—	—	—	—
*Euphorbia heterophylla *L.	100 50	—	—	—	—	—	—	—	—	—	—	—
*Lepidium ruderale *L.	100 50	—	—	—	—	—	—	—	—	—	—	—
*Melanthera latifolia* (Gardner) Cabr.	100 50	—	—	—	—	—	—	—	—	—	—	—
*Mollugo verticillata* L.	100 50	—	—	—	—	—	—	—	—	—	—	—
*Parthenium hysterophorus *L.	100 50	9	10	7 7	9	—	—	—	—	—	—	—
*Ruellia asperula *(Mart. & Nees) Benth. & Hook.	100 50	10	—	—	—	—	—	—	—	—	—	—
*Ruellia geminiflora *Kunth	100 50	—	—	—	8	—	—	—	—	—	—	—
*Sida urens* L.	100 50	20 20	20 20	18 15	—	—	—	—	—	—	—	—
*Spermacoce verticillata *L.	100 50	10 10	8 6	10 7	—	6	—	—	—	—	—	—
*Stylosanthes scabra * Vogel	100 50	8 8	—	—	—	—	—	—	—	—	—	—
*Talinum triangulare *(Jacq.) Willd.	100 50	—	—	—	—	—	—	—	—	—	—	—
Species 1 (Malvaceae)		—	—	—	—	—	—	—	—	30	30	30
Indirect ethnopharmacological approach												
*Alternanthera brasiliana *(L.) Kuntze	100 50	—	—	—	—	—	—	—	—	—	—	—
*Alternanthera tenella* Colla	100 50	—	—	—	—	—	—	—	—	—	—	—
*Commelina erecta *L.	100 50	—	—	—	—	—	—	—	—	—	—	—
*Commelina obliqua Vahl *	100 50	—	—	—	—	—	—	—	—	—	—	—
*Hypenia brachystachys* (Pohl ex Benth.) Harley	100 50	—	—	—	—	—	—	—	—	—	—	—
*Hypenia salzmannii *(Benth.) Harley	100 50	14 12	—	12 12	11 9	—	—	—	—	—	—	—
*Polygala paniculata* L.	100 50	—	—	—	—	—	—	—	—	—	—	—
*Polygala violacea* Aubl.	100 50	—	—	—	—	—	—	—	—	—	—	—
*Portulaca elatior *Mart. ex Rohrb.	100 50	—	—	—	—	—	—	—	—	—	—	—
*Portulaca oleracea* L.	100 50	—	—	—	—	—	—	—	—	—	—	—
*Solanum agrarium *Sendtn.	100 50	—	—	—	—	—	—	—	—	—	—	—
*Solanum americanum *Mill.	100 50	—	—	—	—	—	—	—	—	—	—	—
*Tillandsia recurvata *(L.) L.	100 50	17 15	18 17	18 16	13 13	15 11	—	—	—	—	—	—
*Tillandsia usneoides *(L.) L.	100 50	—	—	—	—	—	—	—	—	—	—	—
Direct ethnopharmacological approach												
*Acalypha multicaulis *Mull. Arg	100 50	12 10	10 10	10 8	—	—	—	—	—	—	—	—
*Acanthospermum hispidum* DC.	100 50	22 20	19 15	19 15	17 15	—	—	—	—	—	—	—
*Ageratum conyzoides *L.	100 50	—	—	—	—	—	—	—	—	—	—	—
*Aosa rupestris* Gardner	100 50	—	—	—	—	—	—	—	—	—	—	—
*Argemone mexicana L. *	100 50	8 7	7 7	7 7	—	—	—	—	—	—	15 15	—
*Boerhavia diffusa* L.	100 50	—	—	—	—	—	—	—	—	—	—	—
*Euphorbia hyssopifolia* L.	100 50	20 16	16 13	18 15	19 16	6 0	—	—	—	—	—	—
*Conocliniopsis prasiifolia *(DC.) R.M. King & H. Rob.	100 50	—	—	—	—	—	—	—	—	—	—	—
*Heliotropium indicum *L.	100 50	—	—	—	—	—	—	—	—	—	—	—
*Hyptis suaveolens *(L.) Poit.	100 50	18 16	15 13	18 16	15 12	—	—	—	—	—	—	—
*Indigofera suffruticosa *Mill.	100 50	30 30	22 22	22 20	—	30 30	—	—	—	—	—	—
*Leonotis nepetifolia *(L.) R. Br.6	100 50	14 13	—	—	—	—	—	—	—	—	—	—
*Ludwigia octovalvis *(Jacq.) P.H. Raven	100 50	13 11	13 12	—	—	—	—	—	—	15 15	13 12	13 12
*Melochia tomentosa* L.	100 50	10 10	14 11	8 7	8 7	11 10	—	—	—	—	—	—
*Momordica charantia L. *	100 50	20 17	15 15	15 14	18 15	18 15	—	—	—	—	—	—
*Physalis angulata *L.	100 50	—	—	—	—	—	—	—	—	—	—	—
*Rhaphiodon echinus *Schauer	100 50	—	—	—	—	—	—	—	—	—	—	—
*Richardia grandiflora *(Cham. & Schltdl.) Steud.	100 50	—	—	—	—	—	—	—	—	—	—	—
*Scoparia dulcis *L.	100 50	12 12	—	—	12 12	—	—	—	—	—	—	—
*Waltheria indica* L.	100 50	—	—	—	—	—	—	—	—	—	—	—

*C*: concentration; Sa: *Staphylococcus aureus*; Se: *S. epidermidis*; Ss: *S. saprophyticus*; Bs: *Bacillus subtilis*; Ef: *Enterococcus faecalis*; Ec: *Escherichia coli*; Pa: *Pseudomonas aeruginosa*; Kp: *Klebsiella pneumonia*; Ca: *Candida albicans*; Ck: *C. krusei* e; Ct: *C. tropicalis*; —: no inhibition.

**Table 2:** Minimal inhibitory concentration (*μ*g/mL) of herbaceous species from the semiarid region, Northeast of Brazil, based on random, direct, and indirect ethnopharmacological approaches.

Species	Sa	Se	Ss	Bs	Ef	Ca	Ck	Ct
Random approach								
*Blainvillea acmella* (L.) Philipson	>1000	>1000	>1000	>1000	NT	NT	NT	NT
*Centratherum punctatum* Cass.	>1000	>1000	>1000	>1000	>1000	NT	NT	NT
*Sida urens* L.	500	1000	1000	NT	NT	NT	NT	NT
Species 1 (Malvaceae)	NT	NT	NT	NT	NT	31.25	62.5	62.5

Indirect ethnopharmacological approach								
*Tillandsia recurvata *(L.) L.	>1000	>1000	>1000	>1000	>1000	NT	NT	NT
*Hypenia salzmannii *(Benth.) Harley	>1000	NT	NT	NT	NT	NT	NT	NT

Direct ethnopharmacological approach								
*Acanthospermum hispidum* DC.	1000	>1000	>1000	>1000	NT	NT	NT	NT
*Argemone mexicana* L.	NT	NT	NT	NT	NT	NT	>1000	NT
*Euphorbia hyssopifolia *L.	1000	>1000	>1000	1000	NT	NT	NT	NT
*Hyptis suaveolens *(L.) Poit.	>1000	>1000	>1000	NT	NT	NT	NT	NT
*Indigofera suffruticosa *Mill.	500	>1000	>1000	1000	>1000	NT	NT	NT
*Leonotis nepetifolia *(L.) R. Br.	>1000	NT	NT	NT	NT	NT	NT	NT
*Ludwigia octovalvis *(Jacq.) P.H. Raven	250	500	NT	NT	NT	125	1000	1000
*Melochia tomentosa* L.	>1000	>1000	NT	>1000	NT	NT	NT	NT
*Momordica charantia L. *	1000	>1000	>1000	1000	>1000	NT	NT	NT

Sa: *Staphylococcus aureus*; Se: *S. epidermidis*; Ss: *S. saprophyticus*; Bs: *Bacillus subtilis*; Ef: *Enterococcus faecalis*; Ca: *Candida albicans*; Ck: *C. krusei* e; Ct: *C. tropicalis*; NT: extract not tested for the strain.
